# Supplementary material for: Association of Self-Reported Sleep Characteristics and Hip Fracture: Observational and Mendelian Randomization Studies
Source: Healthcare (Basel). 2023 Mar 23;11(7):926. doi: 10.3390/healthcare11070926 (PMC10094697; doi:10.3390/healthcare11070926)
Supplement: Supplementary file 1 [file healthcare-11-00926-s001.zip › healthcare-2246527-supplementary.pdf]

Supplementary

Supplementary Figure S1 Scatter plot of the effect of sleep duration on hip fracture in an MR analysis

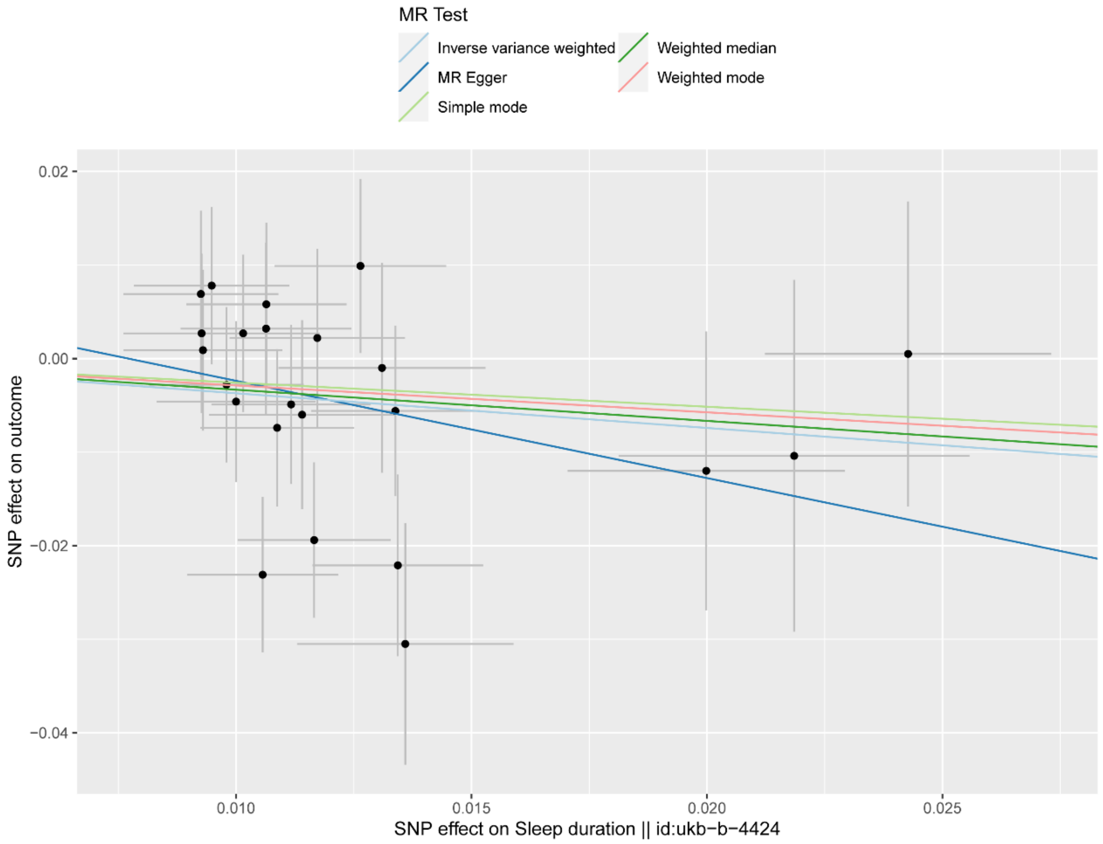

Supplementary Figure S2 Funnel plot of the effect of sleep duration on hip fracture in an MR analysis

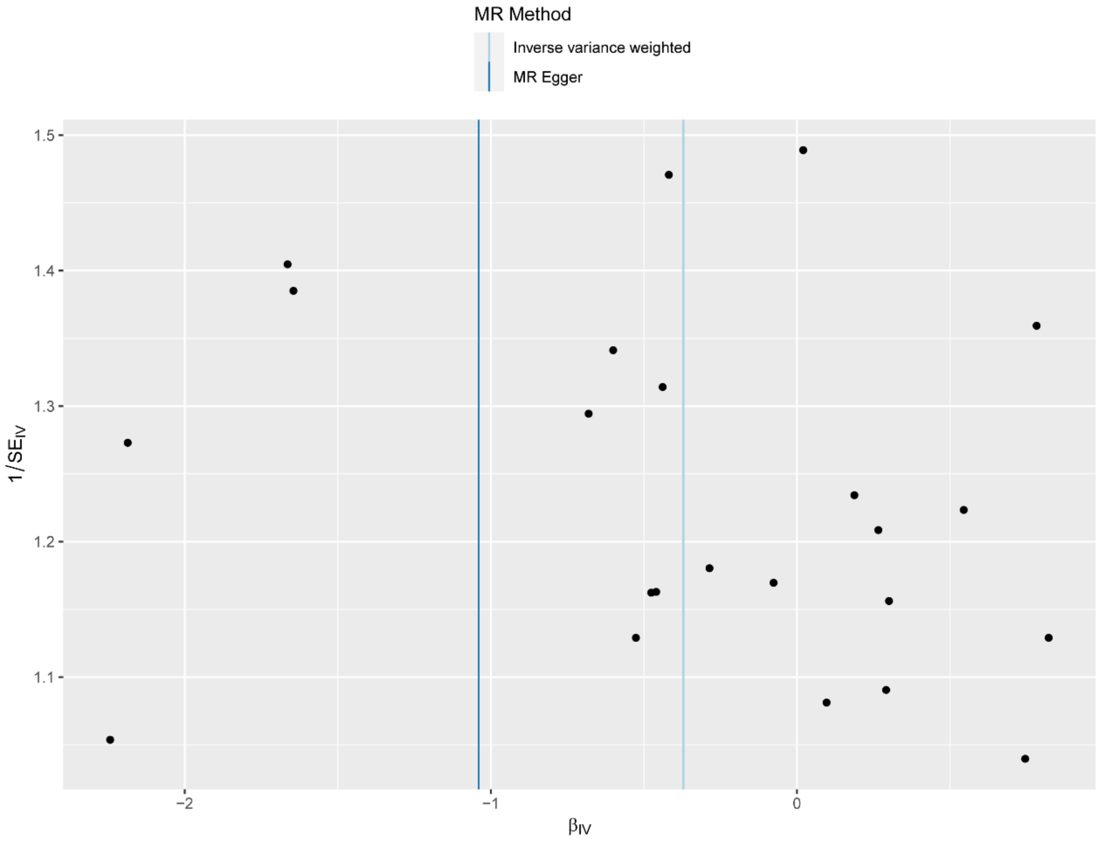

Supplementary Figure S3 Leave-one-out plot of the effect of sleep duration on hip fracture in an MR analysis

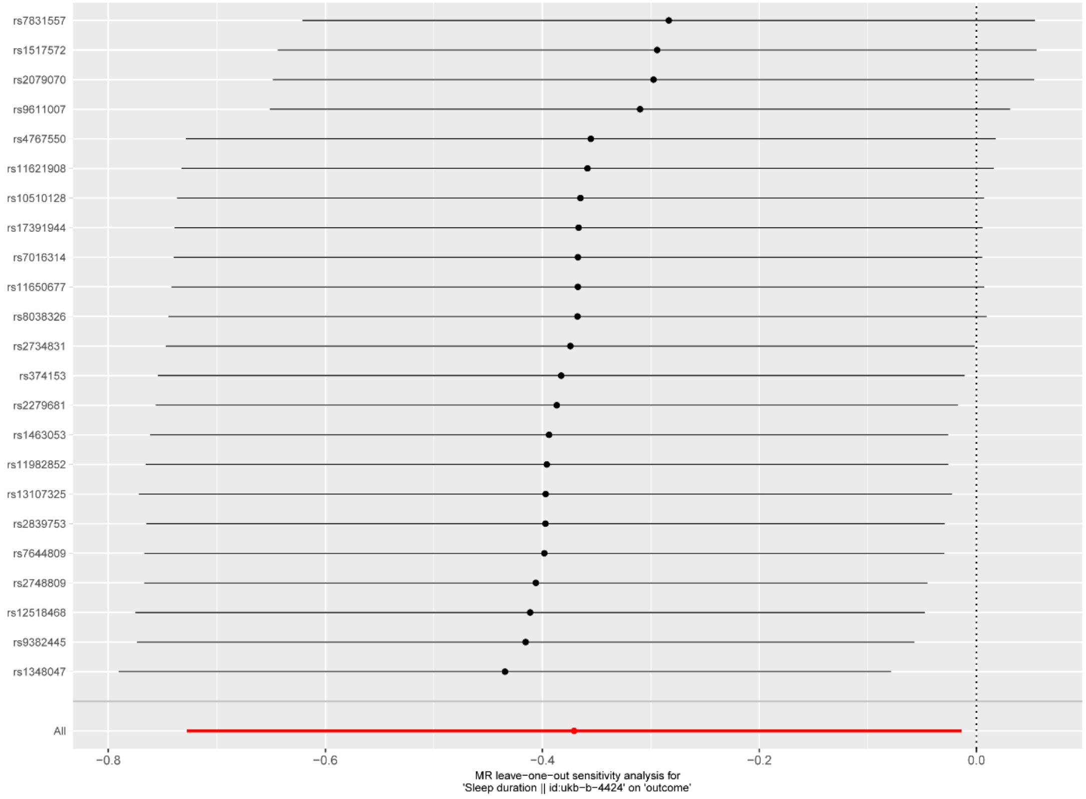

Supplementary Table S1 SNPs site information for Mendelian randomization analysis was performed

SNP	pos.exposure	chr.exposure	effect_allele.exposure	other_allele.exposure	se.exposure	beta.exposure	pval.exposure	samplesize.exposure	eaf.exposure
rs915416	34731984	1	G	C	0.00176	-0.01273	4.80E-13	460099	0.70906
rs2186122	66470206	1	T	A	0.001621	-0.01083	2.30E-11	460099	0.559911
rs2279681	2.02E+08	1	G	C	0.001685	0.009299	3.40E-08	460099	0.341785
rs7517981	31555056	1	C	T	0.001634	-0.00997	1.10E-09	460099	0.601498
rs12567114	98527951	1	A	G	0.001794	0.012338	6.10E-12	460099	0.276368
rs1463053	8705037	1	A	G	0.001661	0.00927	2.40E-08	460099	0.639694
rs6681755	71703970	1	A	G	0.002005	0.011527	9.00E-09	460099	0.199783
rs374153	40382712	2	T	C	0.002197	-0.0131	2.50E-09	460099	0.842567
rs2863957	1.14E+08	2	A	C	0.001929	0.028904	9.60E-51	460099	0.220537
rs1972712	1.67E+08	2	C	T	0.001848	0.011796	1.70E-10	460099	0.249455
rs72831782	1.04E+08	2	A	T	0.001844	-0.01018	3.40E-08	460099	0.269409
rs2683630	58058069	2	G	C	0.001656	0.014951	1.70E-19	460099	0.629076
rs75539574	58871658	2	C	A	0.002874	0.023665	1.80E-16	460099	0.085774
rs35662245	1.48E+08	2	A	T	0.001691	0.010157	1.90E-09	460099	0.338713
rs6783516	1.79E+08	3	T	G	0.001631	-0.00984	1.60E-09	460099	0.583817
rs76258078	55823890	3	G	A	0.003681	-0.02169	3.80E-09	460099	0.049994
rs113021516	1.36E+08	3	C	G	0.001697	0.011481	1.30E-11	460099	0.335898
rs17732997	70470834	3	G	C	0.001618	-0.00884	4.60E-08	460099	0.429679
rs9810474	74961196	3	T	C	0.001894	-0.01115	3.90E-09	460099	0.232184

rs7644809	1.08E+08	3	C	T	0.001625	-0.01015	4.10E-10	460099	0.576033
rs13107325	1.03E+08	4	T	C	0.003039	-0.02427	1.40E-15	460099	0.074905
rs2192528	18327896	4	G	A	0.001601	-0.00981	9.10E-10	460099	0.522493
rs2839753	82287250	4	C	T	0.001812	-0.01064	4.40E-09	460099	0.265347
rs7711696	1.35E+08	5	T	G	0.001735	-0.00987	1.30E-08	460099	0.304987
rs12518468	7249696	5	C	T	0.001703	-0.01064	4.10E-10	460099	0.328685
rs365663	1428883	5	G	A	0.00161	-0.00928	8.10E-09	460099	0.45495
rs6889592	1.02E+08	5	A	G	0.001697	0.011766	4.10E-12	460099	0.332608
rs151014368	1.77E+08	5	A	G	0.001988	0.011379	1.00E-08	460099	0.20734
rs9382445	54937974	6	C	T	0.001649	-0.00948	8.90E-09	460099	0.375168
rs113113059	43160375	6	C	T	0.001933	-0.01113	8.60E-09	460099	0.219834
rs7740402	1.31E+08	6	G	T	0.001735	-0.00951	4.10E-08	460099	0.3061
rs9345234	93162639	6	C	A	0.001624	0.009192	1.50E-08	460099	0.57786
rs34556183	28584775	6	G	A	0.001782	-0.01335	6.60E-14	460099	0.279888
rs11982852	1.33E+08	7	T	C	0.001862	-0.01173	3.10E-10	460099	0.243858
rs62444917	2107262	7	C	A	0.001926	0.012963	1.70E-11	460099	0.222315
rs2079070	1.14E+08	7	G	C	0.001811	-0.01344	1.20E-13	460099	0.734582
rs7831557	10280228	8	A	G	0.001601	-0.01057	4.20E-11	460099	0.517438
rs7016314	14043662	8	C	T	0.001688	0.010001	3.10E-09	460099	0.655927
rs17391944	36901494	9	G	T	0.003724	0.021852	4.40E-09	460099	0.04986
rs112100783	70541193	10	A	G	0.004549	-0.02529	2.70E-08	460099	0.033439
rs10510128	1.25E+08	10	A	G	0.001974	0.011403	7.70E-09	460099	0.207951

rs2236295	64564892	10	T	G	0.001636	-0.00908	2.90E-08	460099	0.403043
rs1517572	28829882	11	C	A	0.001622	0.011659	6.50E-13	460099	0.581256
rs7115856	43769287	11	C	A	0.001603	0.010819	1.50E-11	460099	0.461273
rs11039216	47406592	11	T	C	0.001604	-0.01028	1.50E-10	460099	0.532944
rs2734831	1.13E+08	11	G	T	0.001639	-0.0098	2.20E-09	460099	0.606881
rs174564	61588305	11	G	A	0.001678	0.009745	6.30E-09	460099	0.348602
rs1939455	1.02E+08	11	T	G	0.002517	-0.0158	3.50E-10	460099	0.120291
rs1553132	88297740	11	G	A	0.001825	0.010526	8.10E-09	460099	0.258638
rs34354917	38764559	12	A	C	0.001768	-0.01002	1.40E-08	460099	0.288625
rs4767550	1.18E+08	12	G	A	0.001633	0.010873	2.70E-11	460099	0.413171
rs6561715	53888526	13	A	T	0.001661	0.009782	3.90E-09	460099	0.630636
rs55658675	65554638	14	T	C	0.001675	-0.00969	7.10E-09	460099	0.352906
rs11621908	78495761	14	T	C	0.002943	-0.01999	1.10E-11	460099	0.082807
rs2748809	99735233	14	C	T	0.001645	-0.00925	1.90E-08	460099	0.429258
rs8038326	47989799	15	G	A	0.001793	-0.01338	8.40E-14	460099	0.273169
rs56367859	6540795	16	G	A	0.001636	0.011622	1.20E-12	460099	0.397585
rs9302680	56269892	16	A	G	0.001611	0.012044	7.60E-14	460099	0.439272
rs11643715	23909538	16	G	C	0.00176	0.010949	5.00E-10	460099	0.292625
rs8047587	53798622	16	T	G	0.001613	-0.01102	8.30E-12	460099	0.439514
rs72771082	20023601	16	G	A	0.001936	0.010974	1.40E-08	460099	0.217832
rs8074498	79954544	17	A	T	0.001634	-0.00933	1.10E-08	460099	0.580951
rs11650677	11231513	17	A	G	0.00169	0.01117	3.80E-11	460099	0.339185

rs9903898	45575354	17	T	C	0.001601	-0.00945	3.60E-09	460099	0.488883
rs8072993	21335627	17	G	T	0.001992	0.010922	4.20E-08	460099	0.635677
rs1348047	53050058	18	T	G	0.00182	-0.01264	3.80E-12	460099	0.267253
rs35126035	34986767	19	C	A	0.001644	-0.0092	2.20E-08	460099	0.55833
rs34786000	9942123	19	T	G	0.001628	0.010958	1.70E-11	460099	0.553361
rs2072727	43538733	20	C	T	0.001614	-0.00927	9.30E-09	460099	0.56459
rs9611007	39077058	22	T	C	0.002297	-0.01359	3.30E-09	460099	0.141673
